# Understanding Pressure Effects on Structural, Optical, and Magnetic Properties of CsMnF_4_ and Other 3d^*n*^ Compounds

**DOI:** 10.1021/acs.inorgchem.4c00599

**Published:** 2024-07-10

**Authors:** Guillermo Santamaría, Toraya Fernández-Ruiz, Juan María García-Lastra, Pablo García-Fernández, Inés Sánchez-Movellán, Miguel Moreno, José Antonio Aramburu

**Affiliations:** ● Departamento de Ciencias de la Tierra y Física de la Materia Condensada, Universidad de Cantabria, Avenida de los Castros s/n, 39005 Santander, Spain; ‡ Donostia International Physics Center (DIPC), 20018 Donostia, Euskadi, Spain; § Laboratory for Chemistry of Novel Materials, University of Mons, 7000 Mons, Belgium; ∥ Department of Energy Conversion and Storage, Technical University of Denmark, Anker Engelunds Vej. Building 301, 2800 Kgs. Lyngby, Denmark

## Abstract

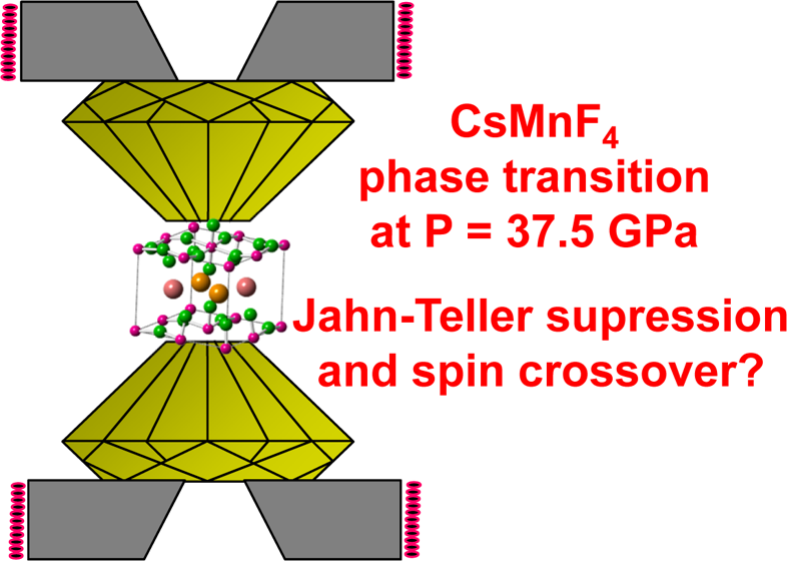

The pressure dependence of structural, optical, and magnetic properties of the layered compound CsMnF_4_ are explored through first-principles calculations. The structure at ambient pressure does not arise from a Jahn–Teller effect but from an orthorhombic instability on MnF_6_^3–^ units in the tetragonal parent phase, while there is a *P*4/*n* → *P*4 structural phase transition at *P* = 40 GPa discarding a spin crossover transition from *S* = 2 to *S* = 1. The present results reasonably explain the evolution of spin-allowed d–d transitions under pressure, showing that the first transition undergoes a red-shift under pressure following the orthorhombic distortion in the layer plane. The energy of such a transition at zero pressure is nearly twice that observed in Na_3_MnF_6_ due to the internal electric field and the orthorhombic distortion also involved in K_2_CuF_4_. The reasons for the lack of orthorhombic distortion in K_2_MF_4_ (M = Ni, Mn) or CsFeF_4_ are also discussed in detail. The present calculations confirm the ferromagnetic ordering of layers in CsMnF_4_ at zero pressure and predict a shift to an antiferromagnetic phase for pressures above 15 GPa consistent with the reduction of the orthorhombicity of the MnF_6_^3–^ units. This study underlines the usefulness of first-principles calculations for a right interpretation of experimental findings.

## Abstract 2

Accurate ab initio calculations on CsMnF_4_ layered material show that the phase transition detected at 37.5 GPa by optical absorption is not the first known case of fluoride complexes with spin crossover from *S* = 2 to *S* = 1. Moreover, contrary to what is assumed in all the literature on this system, our calculations rule out the existence of a Jahn−Teller effect. The origin of the orthorhombic distortion of the complexes is also analyzed.

## Introduction

Insulating transition metal (TM) compounds are an important family of materials characterized by the presence of localized d electrons with strong correlation, giving rise to the interplay of electronic, charge, spin, and orbital degrees of freedom. These compounds are involved in a number of technological applications such as solid-state lasers,^1−3^ devices with colossal magnetoresistance,^4^ or solar cells^5^ and in their understanding appear concepts like orbital ordering, superexchange, or vibronic instabilities.^6−9^

Due to the open shell structure of the cation, TM compounds usually exhibit optical response in the V–UV domain and magnetic ordering, and thus, optical and magnetic tools are widely used in their characterization. In this realm the application of high pressures on a TM compound opens a new window for detecting attractive phenomena such as phase transitions or changes in electronic states of TM complexes where active electrons are localized.^10−15^ In particular, pressure can change the ground-state spin of a complex shifting from a high- to a low-spin configuration^10^ such as happens for a variety of Fe^2+^ complexes.^16^

To understand optical data under pressure, it is crucial to know the evolution of interatomic distances and the nature of involved optical transitions. This requirement is however more difficult to fulfill when the TM complex is distorted from octahedral symmetry. As both conditions are often not fulfilled, first-principles calculations can be of help for gaining the right insight into this matter. Furthermore, in high-pressure experiments optical spectra are sometimes only recorded at room temperature^12,17^ where the resolution is certainly poorer than at 4.2 K, and thus, theoretical calculations can aid to overcome that hindrance as well.

This work is devoted, in a first step, to understand the optical absorption spectra under pressure of CsMnF_4_.^17^ This compound belongs to the interesting family of fluoromanganates^18−20^ with formula AMnF_4_ (A = alkali monocation or NH_4_^+^) involving the 3d^4^ cation Mn^3+^. This family has deserved much attention as a model to correlate the various structural, magnetic, and optical properties, both at ambient pressure (study of the chemical pressure effects linked to the change of monocation A) and under high hydrostatic pressure. Among these layered compounds the most studied system is just CsMnF_4_ as it is the only one with a tetragonal structure and ferromagnetic (FM) order in the layer planes at ambient pressure when *T* < *T*_c_ = 23 K.^19^

**Figure 1:**
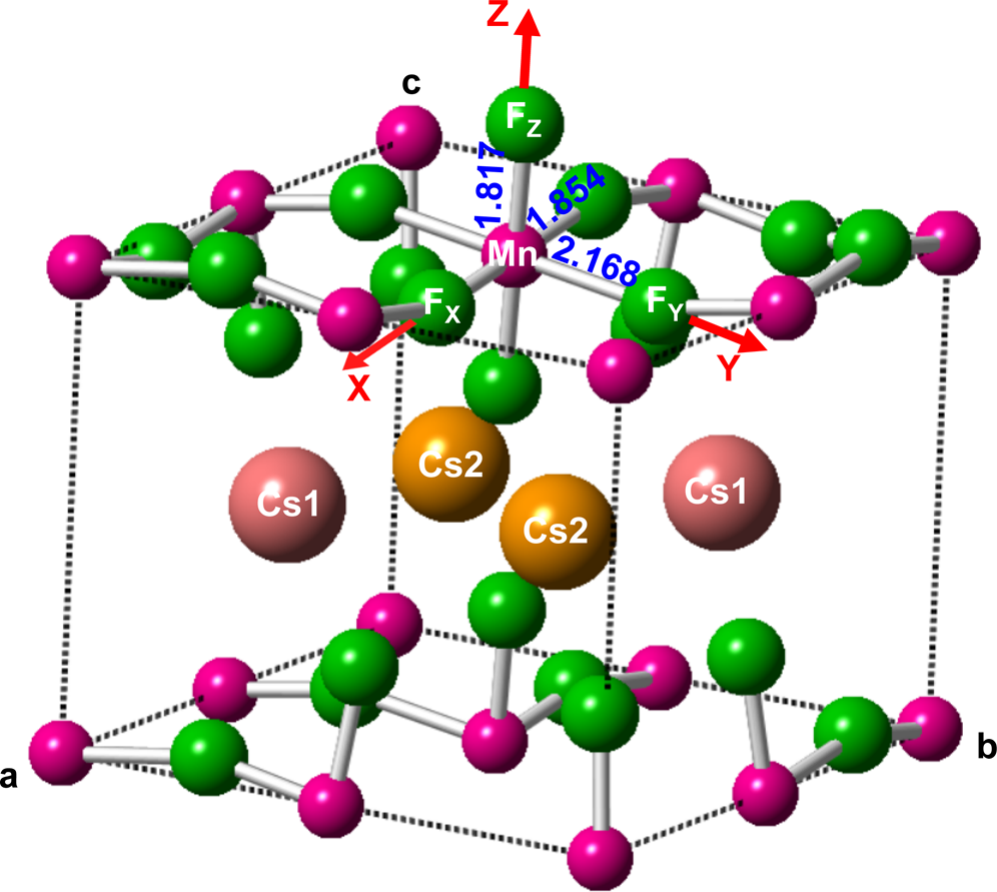
Experimental unit cell of the CsMnF_4_ structure corresponding to the *P*4/*n* phase at *P* = 0. Red arrows indicate the local {X, Y, and Z} axes of a MnF_6_^3–^ complex. Blue numbers indicate the Mn–F distances (in Å). The lattice parameters are *a* = *b* = 7.944 Å and *c* = 6.338 Å.

According to X-ray diffraction data by Molinier and Massa, CsMnF_4_ at ambient pressure belongs to the *P*4/*n* space group.^18^ The structure is depicted in Figure 1 showing that Mn^3+^ ions are disposed in layers in the *ab* plane although the F^–^ ions of MnF_6_^3–^ units are not strictly in that plane just reflecting the existence of buckling. In the involved MnF_6_^3–^ units the shortest Mn–F distance corresponds to the *z* direction, perpendicular to the *ab* layer plane (*R*_*z*_ = 1.817 Å), while the longest one (*R*_*y*_ = 2.168 Å) is nearly perpendicular to the *z* direction. The last Mn–F distance (*R*_*x*_ = 1.854 Å) differs from that of *R*_*z*_ by only 0.037 Å.

Interestingly, the F–Mn–F angle of MnF_6_^3–^ units in CsMnF_4_ differs by less than 3° from 90°. This fact and a Mn–F–Mn angle of 162°, a consequence of buckling, leads to a local symmetry around a Mn^3+^ ion that is not strictly orthorhombic (*D*_2*h*_) but *C*_*i*_. CsMnF_4_ exhibits an antiferrodistortive arrangement and thus in two adjacent MnF_6_^3–^ units that share a common ligand the longest axes are essentially perpendicular. The RbMnF_4_ and KMnF_4_ compounds of the AMnF_4_ family also involve puckered layers but do not belong to the *P*4/*n* space group and have a Mn–F–Mn angle equal to 148° and 140°, respectively.^18,19^ Both compounds are antiferromagnetic but only below 5 K.^19,20^

**Figure 2:**
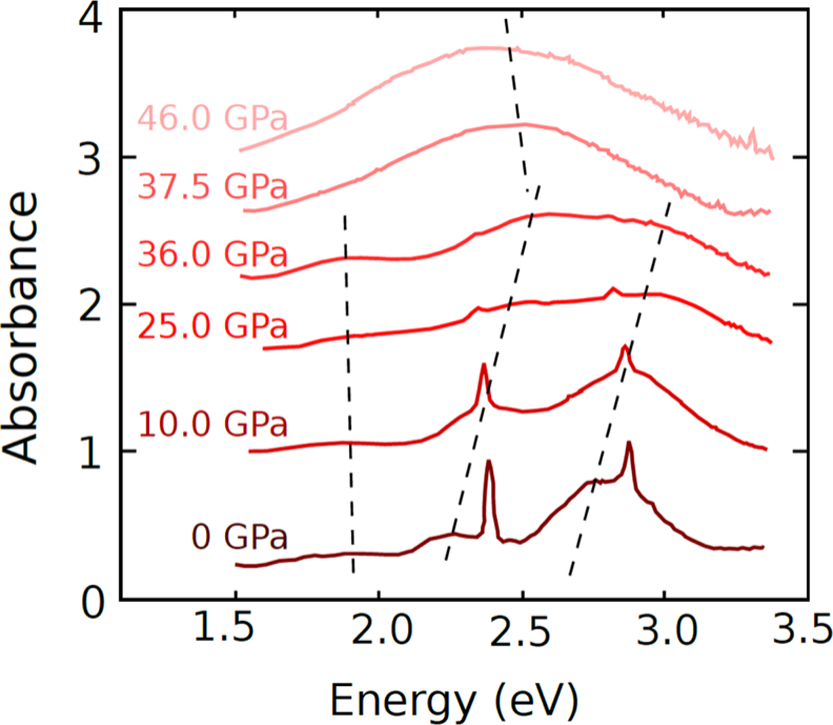
Experimental optical spectra of CsMnF_4_ in the 1.5–3.5 eV range measured at room temperature for zero pressure and also *P* = 10.0, 25.5, 36.0, 37.5, and 46.0 GPa, (adapted from ref (17)). Dotted lines are the approximate variations in the energies of the band maxima as proposed in ref (17), although this proposal is discussed in the text.

In the interpretation of structural and optical data (Figure 2) of CsMnF_4_ there are two relevant questions that need to be clarified: (1) The local distortion of MnF_6_^3–^ units in CsMnF_4_ has usually been ascribed to the Jahn–Teller effect^17−21^ despite the low symmetry of the compound. (2) From the optical absorption data under pressure (Figure 2) it has been proposed that the ground state of MnF_6_^3–^ units in CsMnF_4_ undergoes a spin crossover transition from *S* = 2 to *S* = 1 for a pressure around 38 GPa.^17^ As these kinds of transitions have been observed for complexes with ligands such as CN^–^ or SCN^–^^10,16^ its possible existence for a fluoride complex certainly deserves a further investigation. For achieving this goal, the lack of data on the evolution of Mn–F distances under pressure for CsMnF_4_ hampers the interpretation of optical absorption results, a circumstance that can, however, be overcome by means of theoretical calculations.

Finally, the present work also pays attention to the ferromagnetism displayed by CsMnF_4_ at zero pressure, as well as the evolution of the magnetic order under an applied pressure. At this point, the recent results derived for insulating layered compounds like K_2_CuF_4_ or Cs_2_AgF_4_ shed light on that issue.^9,22,23^

This work is organized as follows. The computational tools employed in this work are briefly described in the next section, and we then discuss the previous interpretations of experimental data on CsMnF_4_ together with the main results obtained in this work. Some final remarks are reported in the last section.

## Computational Tools

First-principles density functional theory (DFT) calculations (performed using hybrid exchange–correlation functionals) were conducted to analyze the influence of the pressure on the geometry, magnetic order, and optical transitions of the layered compound CsMnF_4_. In order to be sure of the calculated properties, we have used two codes, Crystal17 and VASP, with different implementations of the DFT for periodic crystals, obtaining very similar results.

On the one hand, the Crystal17 code^24^ has two relevant implementations, significantly speeding up the calculations: first, it makes full use of the symmetries of the material’s space group, and second, Bloch orbitals are represented through linear combinations of Gaussian type functions.^25−27^ On the other hand, Vienna Ab initio Simulation Package (VASP)^28,29^ employs a set of plane waves to describe the Bloch orbitals, enabling highly accurate results in reproducing experimental geometries in all kinds of compounds, including organic and hybrid organic–inorganic materials.^30−33^

First, we optimized with the Crystal17 code the geometry of CsMnF_4_ at the experimental *P*4/*n* phase^18^ using different magnetic phases, obtaining the minimum energy for the experimental FM order with values of both lattice parameters and bond Mn–F distances similar to the experimental data, with discrepancies of less than 3.5%. The next step was the acquisition of the parent phase of the experimental CsMnF_4_*P*4/*n* structure. For this goal we started from the experimental CsMnF_4_ structure substituting all open shell Mn^3+^ ions, with d^4^ electronic configuration and *S* = 2, by Fe^3+^ ions (d^5^ electronic configuration and *S* = 5/2) with spherical density (in vacuo) and equal ionic radius, *r*(Mn^3+^) ≈ *r*(Fe^3+^) ≈ 0.785 Å.^34^ The symmetry of the optimized CsFeF_4_ parent phase was *P*4/*nmm*, precisely the experimental phase of this compound.^35^ Moreover, in order to elucidate the nature of the phase transition experimentally observed in CsMnF_4_ under a pressure *P* ≈ 38 GPa,^17^ we have also optimized the structure of this compound under hydrostatic pressures in the range of *P* = 0–60 GPa. Furthermore, for each optimized structure, we have followed the unstable harmonic modes in the supercell and found the corresponding ground state.

Geometry optimizations under pressure from 0 to 60 GPa, in 10 GPa increments, were also performed with the VASP code using initial geometries provided by the Crystal17 optimizations, significantly expediting the VASP convergence process.

Energies of the d–d optical transitions were calculated for each optimized geometry under pressure. For the Crystal17 optimized geometries, DFT calculations have been carried out on MnF_6_^3–^ complexes by means of the Amsterdam density functional (ADF) code.^36,37^ In these pseudomolecular calculations, the MnF_6_^3–^ clusters were embedded in the electrostatic potential of the rest of lattice ions,^38^ which was previously calculated through Ewald–Evjen summations.^39,40^

In a different strategy, we have also calculated the energy values of these d–d transitions with VASP, using a different embedding by substituting three of the four Mn^3+^ atoms in the unit cell with Ga^3+^, atoms with a d^10^ configuration, and thus a symmetric electron density. The energies of the optical transitions were determined for each pressure ranging from 0 to 60 GPa, by using the ΔSCF method.^41,42^ The ΔSCF method consists of a self-consistent calculation of the total energy for the ground state, yielding the corresponding *E*_GS_. The geometries used are the ones obtained from the optimization of the periodic CsMnF_4_ system. At the same geometry the electronic configuration of an excited state is imposed by manually adjusting the occupations of the d-like Kohn–Sham orbitals, and the SCF procedure is performed again, to obtain the corresponding *E*_exc_ energy. The energy difference, *E*_exc_– *E*_GS_, is taken as the electronic transition energy. Results of these calculations are very similar to the ones performed by means of the ADF code.

More details on the calculations with both codes can be found in the Supporting Information.

## Analysis of Previous Interpretations of Structural and Optical Data for CsMnF_4_

The optical properties in the V–UV domain of an insulating compounds like CsMnF_4_ greatly depend on the involved MnF_6_^3–^ units, whose ground state in *O*_*h*_ symmetry can, in principle, be either ^5^E_g_(t^3^e^1^) or ^3^T_1g_(t^4^e^0^).^43^ Nevertheless, experimental data, at ambient pressure, on compounds containing MnF_6_^3–^ support a high-spin configuration (*S* = 2) as ground state.^12,19,20^ Octahedral complexes with inorganic ligands like F^–^ or Cl^–^ display a high-spin configuration for 3d^*n*^ ions (*n* = 5, 6) like Fe^3+^ or Fe^2+^ while a low-spin configuration is found for complexes of 4d and 5d ions, like Ru^3+^ or Ir^4+^, involving higher 10Dq values.^10,44^ A low-spin (*S* = 1/2) ground state has also been reported^45,46^ for NiF_6_^3–^ complexes in A_3_NiF_6_, Cs_2_ANiF_6_ (A = Na, K), and Rb_2_NaNiF_6_ fluorides and also in oxides doped with Ni^3+^.^47^ However, electron paramagnetic resonance (EPR) measurements carried out in Ni^3+^-doped KMgF_3_ and CsCaF_3_ perovskites^48,49^ lead to a high-spin (*S* = 3/2) ground state. These results show that, in NiF_6_^3–^ complexes, the high-spin/low-spin energy separation must be very small.^45,46^ Moreover, magnetic measurements performed on Li_3_NiF_6_ characterized at low temperature a low-spin configuration (*S* = 1/2), which tends to a high-spin one with increasing temperature.^50^ To the best of our knowledge, a spin crossover under pressure in fluorides containing NiF_6_^3–^ complexes has not been found.

As in 6-fold coordinated compounds of d^4^ ions (Mn^3+^, Cr^2+^), the local symmetry of a MnF_6_^3–^ complex corresponds to a distorted octahedron, and the ground state wave function, Ψ_g_, can briefly be written as
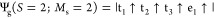
1Here the three t_*i*_ (*i* = 1, 2, 3) and e_*j*_ (*j* = 1, 2) orbitals come from the t_2g_(*xz*, *xy*, *yz*) and e_g_(3*z*^2^–*r*^2^, *x*^2^–*y*^2^) orbitals in *O*_*h*_ symmetry, and thus, the e_2_ orbital is empty in the ground state.^13^ Accordingly, the spin-allowed transitions (Δ*S* = 0) are simply described by t_*i*_↑ → e_2_↑ (*i* = 1, 2, 3) and e_1_↑ → e_2_↑. As the e_1_↑ → e_2_↑ transition is usually the lowest, its energy is termed *E*_0_ while those associated with the t_i_↑ → e_2_↑ transitions are simply called E_*i*_ (*i* = 1, 2, and 3). In addition to the spin-allowed transitions, sharp peaks with smaller oscillator strengths associated with forbidden transitions (Δ*S* = −1) are sometimes observed in optical spectra of d^4^ ions (Mn^3+^, Cr^2+^). They correspond to excited states with *S* = 1 and are described by determinants like |t_1_↑ t_2_↓ t_3_↑ e_1_↑| so the total spin of the t-subshell is only 1/2. Such forbidden transitions have well been detected for CrF_2_.^51^ Similar transitions with Δ*S* = −1 are also observed in the absorption spectra of CrO_6_^9–^ or CrF_6_^3–^ units and are little sensitive to pressure.^52,53,14,15^

Figure 2 reproduces the experimental optical absorption spectra at room temperature of CsMnF_4_ in the 1.5–3.5 eV range for pressures up to 46 GPa.^17^ The spectra for *P* < 25 GPa involve three poorly resolved broad bands and two sharp peaks that are little sensitive to pressure and progressively disappear at higher pressures. Accordingly, it is not easy to know from experimental results at ambient pressure (Figure 2) the number of spin-allowed transitions and the corresponding energies. In the pressure range 37.5–46.0 GPa only one broad band is observed in the optical spectrum of CsMnF_4_ whose maximum is around 2.5 eV, and its bandwidth is higher than ∼1 eV. No signs of a structural phase transition around 1.4 GPa, early suggested by Moron et al.,^21^ have been found in the optical measurements on CsMnF_4_.

Seeking to understand the optical spectra of CsMnF_4_ at ambient pressure it has been proposed that the experimental geometry of MnF_6_^3–^ units is the result of a static Jahn–Teller (JT) effect.^17^ Under that assumption, also followed in other works,^18−21^ the JT effect would be responsible for having tetragonal MnF_6_^3–^ units with Y as the local principal axis of the complex (Figure 1). Such units are elongated in accord with the geometry widely observed for systems actually displaying a static JT effect.^54−56^ Consequently, e_1_ would be a molecular orbital transforming like 3*y*^2^–*r*^2^ while the unoccupied e_2_ orbital corresponds to *z*^2^–*x*^2^. According to this hypothesis, the optical absorption spectrum of CsMnF_4_ at ambient pressure would involve three spin-allowed transitions in the MnF_6_^3–^ unit whose energies have been proposed^17^ to be *E*_0_ = 1.80 eV, *E*_1_ = 2.26 eV, and *E*_2_ = *E*_3_ = 2.80 eV from Figure 2. Such values are close to *E*_0_ = 1.92 eV, *E*_1_ = 2.23 eV, and *E*_2_ = *E*_3_ = 2.73 eV reported by Morón and Palacio.^57^

Nonetheless, doubts are raised by the JT assumption due to the following reasons:^54−57,9^

(i) The existence of a JT effect requires a degenerate electronic state in the *initial* geometry. Therefore, as CsMnF_4_ is a layered compound, even if the geometry of the initial parent phase is tetragonal the electronic ground state of an MnF_6_^3–^ unit should not be degenerate according to symmetry.
(ii) CsMnF_4_ is a layered compound where layers are perpendicular to the crystal **c** axis (Figure 1). Accordingly, one would expect that the axis of the MnF_6_^3–^ unit perpendicular to the layer plane plays a singular role, a fact seemingly not consistent with the JT assumption.
(iii) The local equilibrium geometry for MnF_6_^3–^ in CsMnF_4_ is not tetragonal. Indeed, even assuming Y as the main axis (Figure 1) the symmetry would be at most orthorhombic because *R*_X_ – *R*_Z_ = 0.037 Å. Accordingly, one should expect four and not only three d–d transitions with Δ*S* = 0 for CsMnF_4_.
(iv) Although most of the d^9^ systems which exhibit a static JT effect are elongated with a hole in an *x*^2^–*y*^2^ type orbital^54−57^ this is not a general rule. For instance, in the cubic CaO lattice doped with Ni^+^, the hole is in a 3*z*^2^–*r*^2^ orbital and the octahedron compressed.^58,59^ In non-JT systems like K_2_ZnF_4_:Cu^2+^, where the host lattice is tetragonal, the hole is also in 3z^2^–*r*^2^.^60,61^

Looking at higher pressures, the optical spectrum of CsMnF_4_ seems to undergo some change around 37 GPa as above this pressure the optical spectrum at room temperature involves only one very broad band (Figure 2). Such effect has been assumed to arise from a change of the ground-state configuration which would be ^3^T_1g_(t^4^e^0^) when *P* > 37 GPa with an *O*_*h*_ local geometry for MnF_6_^3–^ units.^17^

No further arguments are given for underpinning that assumption that casts in principle some doubts. Indeed, just looking at Tanabe–Sugano diagrams^43^ one would expect that in octahedral symmetry the transition of a ^5^E_g_(t^3^e^1^) ground state to ^3^T_1g_(t^4^e^0^) in MnF_6_^3–^ takes place for 10Dq ≥ 3 eV. For estimating whether this condition is fulfilled, it is useful to consider that the 10Dq value derived from the four allowed transitions in Na_3_MnF_6_ (10Dq = 1.88 eV) is close to 10Dq measured for octahedral CrF_6_^3–^ units in cubic elpasolites involving also a trivalent TM cation.^14,62,63^ For instance, in the case of Rb_2_KCrF_6_, 10Dq = 1.97 eV at ambient pressure while a value d(10Dq)/d*P* = 0.014 eV/GPa has been measured in the range 0–10 GPa.^63^ Accepting this value for a hypothetical *O*_*h*_ MnF_6_^3–^ unit, one would expect 10Dq = 2.41 eV for *P* = 38 GPa. This figure is thus below 10Dq = 3 eV, which is the estimated value required for the spin crossover.

Given these facts, this work addresses the following questions centered on the interpretation of optical data under pressure in CsMnF_4_: (1) the origin of the local geometry of MnF_6_^3–^ units in CsMnF_4_ and the nature of the electronic ground state at zero pressure; (2) the number and energies of the spin-allowed transitions at zero pressure and its evolution as a function of the applied pressure; and (3) the nature of the phase transition observed around 37 GPa, paying particular attention to a possible shift of the ground state spin from *S* = 2 to *S* = 1.

## Results and Discussion

### Structure and Electronic Ground State at Zero Pressure

The calculated values of the lattice parameters and Mn–F distances of CsMnF_4_ at zero pressure are collected in Table 1. Such values, derived using both CRYSTAL and VASP codes in a *P*4/*n* space group, are compared to experimental findings.^18,19^ As can be seen in Table 1 the differences between calculated and experimental values are always smaller than 1.5%. The electronic ground state of the MnF_6_^3–^ unit is found to come from the ^5^E_g_(t^3^e^1^) configuration in cubic symmetry, thus implying a high-spin configuration with *S* = 2.

**Table 1:** Calculated Lattice Parameters and Mn–F Distances^a^ for CsMnF_4_ at Zero Pressure in the *P*4/*n* Space Group Using VASP (First Row) and CRYSTAL (Second Row) Codes and Compared to Experimental Values^18,19^

	*a* = *b*	*c*	*R*_Z_	*R*_X_	*R*_Y_
Experimental	7.944	6.338	1.817	1.854	2.168
Calculated	8.029	6.401	1.829	1.871	2.189
	7.961	6.347	1.818	1.877	2.161

a All distances are given in Å.

Seeking to understand the origin of the local structure in CsMnF_4_, it is useful to analyze the evolution of the crystal structure when Mn^3+^ is replaced by a cation like Fe^3+^ with a similar ionic radius, giving rise to the so-called high-symmetry parent phase.^6,13,22,23^ Under strict octahedral symmetry, Fe^3+^ in high-spin configuration (*S* = 5/2) exhibits a nearly spherical electronic density which is however never found for a 3d^4^ ion like Mn^3+^ or a 3d^9^ ion like Cu^2+^ in the same situation. After the Mn^3+^ → Fe^3+^ substitution, we carried out a geometry optimization of the CsFeF_4_ structure maintaining fixed the experimental *P*4/*n* space group of CsMnF_4_. The obtained final structure has a higher symmetry belonging to the tetragonal *P*4/*nmm* space group, although it still involves buckled layers (Table 2). It should be noted that the obtained local geometry of each FeF_6_^3–^ unit in CsFeF_4_ corresponds to a compressed octahedron with *R*_X_ = *R*_Y_ and *R*_Z_ < *R*_X_ (Table 2) and a symmetry practically tetragonal with Z as the main axis. Interestingly in the present case the calculated parent phase coincides with the experimental structure of the CsFeF_4_ compound at normal conditions.^64,65^ The calculated lattice parameters and Fe–F distances correspond to experimental ones within 1.5%.

**Table 2:** Calculated Lattice Parameters and Fe–F Distances for CsFeF_4_ at Zero Pressure Assuming, in Principle, the *P*4/*n* Space Group^a^

	*a* = *b*	*c*	*R*_Z_	*R*_X_	*R*_Y_
Calculated	7.926	6.631	1.863	1.989	1.989
Experimental	7.787	6.540	1.884	1.959	1.959

a The results, derived through the CRYSTAL code, show that the system evolves until reaching the *P*4/*nmm* space group, where *R*_X_ = *R*_Y_ due to the higher symmetry. Experimental results^65^ are given for comparison. All distances are given in Å.

**Figure 3:**
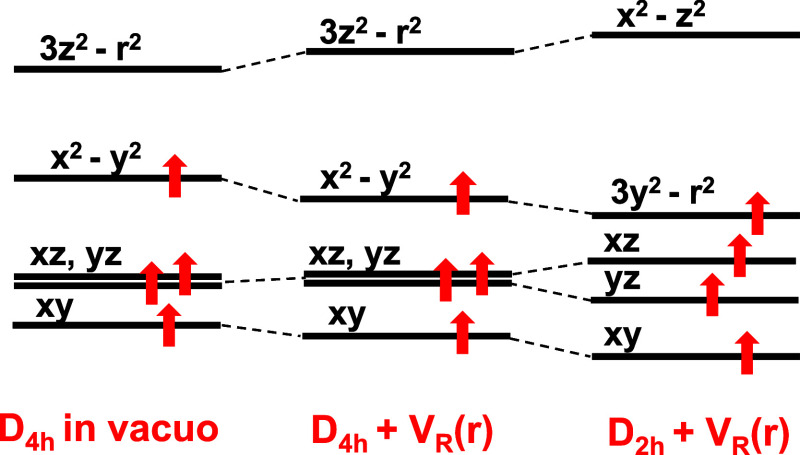
Qualitative scheme of the energy levels of the 5 antibonding orbitals with mainly d character of the MnF_6_^3–^ complex in CsMnF_4_ at zero pressure depicted in 3 steps: (1) complex in vacuo with *D*_4*h*_ geometry, (2) adding the internal V_R_(**r**) potential, and (3) in *D*_2*h*_ geometry and including the V_R_(**r**) potential.

Bearing the preceding considerations in mind, we first consider CsMnF_4_ in the tetragonal *P*4/*nmm* geometry of the parent phase. In that case, the MnF_6_^3–^ units are essentially tetragonal with *R*_Z_ = 1.80 Å and *R*_X_ = *R*_Y_ = 2.011 Å. The main axis is Z, perpendicular to the layer plane, and the four antibonding valence orbitals are ordered as shown in Figure 3, giving rise to an orbitally singlet ground state. Following the compressed geometry, the LUMO corresponds to the molecular orbital of the MnF_6_^3–^ unit transforming like 3*z*^2^–*r*^2^ and is simply designated by |3*z*^2^–*r*^2^⟩ while the highest occupied molecular orbital, HOMO, is |*x*^2^–*y*^2^⟩. Accordingly, the ground state belongs to ^5^A_1g_ in tetragonal symmetry and the |*x*^2^–*y*^2^⟩ → |3*z*^2^–*r*^2^⟩ excited state to ^5^B_1g_.

At this point it is important to highlight that the gap, *E*_0_, associated with the |*x*^2^–*y*^2^⟩ → |3*z*^2^–*r*^2^⟩ excitation in MnF_6_^3–^ does not *only* reflect that in the parent phase *R*_Z_ < *R*_X_ = *R*_Y_. Indeed, although active electrons are essentially localized in the MnF_6_^3–^ unit they also feel the internal electric field, E_R_(**r**), due to ions of CsMnF_4_ lying *outside* the complex, that gives rise to an extrinsic contribution to *E*_0_ such as has been proved for layered compounds like K_2_CuF_4_ or La_2_CuO_4_^6,9,23,61,66^

**Figure 4:**
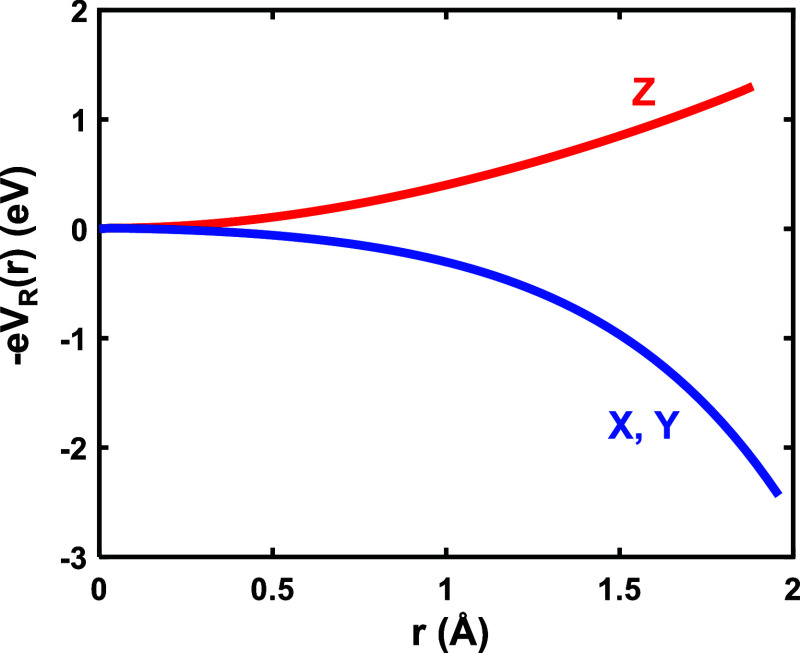
Potential energy (−e)V_R_(**r**) corresponding to the internal electric field created by the rest of the lattice ions of CsMnF_4_ in the metastable *P*4/*nmm* phase of CsFeF_4_ on a MnF_6_^3–^ complex. Energies are depicted along the local X, Y, and Z directions of the complex.

Figure 4 depicts the electrostatic potential V_R_(**r**) associated with the internal field through . According to the shape of (−e)V_R_(**r**) it favors to increase the energy of |3*z*^2^–*r*^2^⟩ and reduce that of |*x*^2^–*y*^2^⟩, thus enhancing the value of *E*_0_. We have derived a value *E*_0_ = 0.7 eV considering only the isolated MnF_6_^3–^ unit while *E*_0_ = 1.2 eV is obtained once V_R_(**r**) is incorporated into the calculation. Therefore, V_R_(**r**) not only helps to stabilize |*x*^2^–*y*^2^⟩ as HOMO but likely has a significant influence on optical transitions, a matter that will be discussed later.

**Figure 5:**
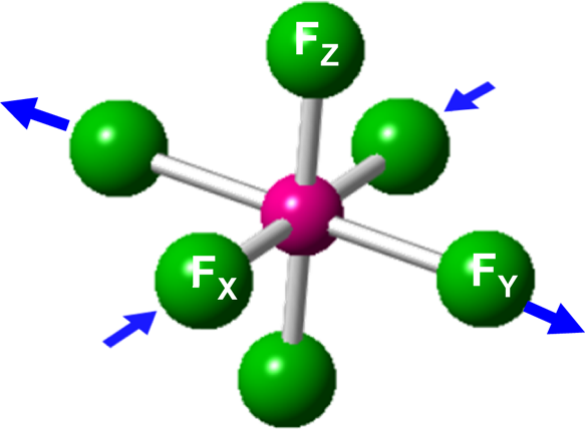
Picture of the local b_1g_ vibrational mode, which is unstable in CsMnF_4_ in the parent phase *P*4/*nmm* at *P* = 0.

Interestingly, if CsMnF_4_ is in the *P*4/*nmm* phase the electronic density in the MnF_6_^3–^ unit with an unpaired electron in |*x*^2^–*y*^2^⟩ is compatible with a tetragonal symmetry (*R*_Z_ < *R*_X_ = *R*_Y_) of the complex. This fact is thus consistent with the lack of JT effect under an *initial* tetragonal symmetry such as happens for K_2_ZnF_4_:Cu^2+^.^61,76^ Nevertheless, the equilibrium geometry of CsMnF_4_ exhibits a lower *P*4/*n* symmetry with *R*_Y_ > *R*_X_ for the MnF_6_^3–^ complex. This instability of the *P*4/*nmm* structure for CsMnF_4_ implies the existence of at least one vibration mode with imaginary frequency.^23^ Accordingly, we have calculated the vibrational frequencies of CsMnF_4_ in the optimized *P*4/*nmm* geometry finding an a_2g_ lattice mode with a frequency equal to 367i cm^–1^. The effect of that mode on the MnF_6_^3–^ unit is described by the orthorhombic b_1g_ local mode (Figure 5) which is thus responsible for having *R*_Y_ – *R*_X_ = 0.31 Å in the final equilibrium geometry of CsMnF_4_ at *P* = 0 (Table 1). The difference, Δ*U*, between the calculated energy per molecule for the equilibrium *P*4/*n* and the parent *P*4/*nmm* phase amounts to 71 meV and is thus the source for the orthorhombic instability shown in Figure 5. That instability, similar to that found for K_2_CuF_4_ or Cs_2_AgF_4_,^9,23^ is driven by the electron–vibration coupling, H_vib_, and involves changes in the ground state wave function and the associated electronic density.^8,67^

If H_0_ denotes the Hamiltonian where all nuclei are frozen at a given position, the Hamiltonian H describing the small motions around it following the distortion coordinate Q of a nondegenerate mode can simply be written as
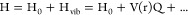
2It should be noted that H_vib_ exhibits the same symmetry as H_0_ provided that symmetry operations are carried out on both electron and nuclei coordinates. Accordingly, in eq 2, V(r) transforms like the coordinate Q, and thus both operators belong to the same Γ irrep. If Ψ_g_(r) is the wave function of the ground-state orbital singlet then ⟨Ψ_g_(r)| V(r)| Ψ_g_(r)⟩ = 0 unless Q refers to the totally symmetric vibration belonging to a_1g_. However, in second-order perturbations H_vib_ can couple Ψ_g_(r) with excited states, Ψ_n_(r), belonging to Γ_n_, giving rise to a *decrement*, ΔE_g_, of the ground-state energy
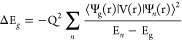
3Thus, the excited states verifying Γ_g_ × Γ_n_ ⊃ Γ can be coupled to the ground state. This fact *modifies* the electronic density and also yields a *negative* contribution, −K_ν_ ,to the total force constant, K, which can be written as
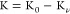
4Here, K_0_ stands for the positive contribution associated with the frozen electronic density of H_0_ while K_ν_ reflects the electronic density changes due to H_vib_ and is given by
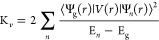
5Thus, the instability responsible for the equilibrium structure of CsMnF_4_ appears because the K_0_ < K_ν_ condition is fulfilled in this case. Interestingly, this implies the admixture of the ^5^A_1g_ ground state of MnF_6_^3–^ with the excited ^5^B_1g_ through a b_1g_ local mode, thus modifying the electronic density of the complex. It is worth noting that, in CsMnF_4_, two adjacent MnF_6_^3–^ units share a common ligand, a fact that is behind the instability developed in K_2_CuF_4_ or Cs_2_AgF_4_^23,9^ but not in K_2_ZnF_4_:Cu^2+^^23,9,60,61^ or even in KAlCuF_6_^68−70^ where the CuF_6_^4–^ units are well-separated. In addition, the parent phase of CsMnF_4_ involves compressed MnF_6_^3–^ units giving rise to softer bonds in the layer plane, a fact that also helps to develop the orthorhombic instability such as has previously been discussed.^23,9,22^

In the equilibrium geometry of CsMnF_4_ at zero pressure the highest occupied molecular orbital (HOMO) wave function of the MnF_6_^3–^ unit, |φ_H_⟩, is not purely |*x*^2^–*y*^2^⟩ but involves an admixture of |3*z*^2^–*r*^2^⟩ as a result of the symmetry reduction due to the instability
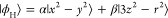
6The present calculations yield α^2^ = 85%, stressing that the HOMO wave function keeps a dominant |*x*^2^–*y*^2^⟩ character once the distortion takes place and *R*_Y_ > *R*_X_. A similar situation has been found in other layered systems like K_2_CuF_4_ or Cs_2_AgF_4_.^23,9^ Interestingly, if we write eq 6 using the {|*x*^2^–*z*^2^⟩, |3*y*^2^–*r*^2^⟩} basis, then
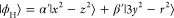
7If α^2^ = 85%, it is simple to find β′^2^ = 98% demonstrating that the HOMO wave function is essentially |3y^2^–*r*^2^⟩ and thus greatly localized along the longest Y axis. In the same way, the LUMO wave function, |φ_L_⟩, is basically equal to |*x*^2^–*z*^2^⟩ despite the electronic structure of CsMnF_4_ not being due to the JT effect.

For the sake of completeness, a qualitative picture of the electronic ground state of MnF_6_^3–^ at the equilibrium geometry of CsMnF_4_ at zero pressure is shown on Figure 3. Accordingly, the lowest d–d excitation is simply described by |3*y*^2^–*r*^2^⟩ → |*x*^2^–*z*^2^⟩. This matter will be discussed later.

### Structural Changes Induced by Pressure

Results of calculations on CsMnF_4_ under applied pressure were performed using both CRYSTAL and VASP codes. By means of them, we can derive the enthalpy per molecule, H = U + PV, responsible for the equilibrium structure at *T* = 0 K. Below *P* = 40 GPa the equilibrium structure of CsMnF_4_ is always found to be described by the space group at ambient pressure (*P*4/*n*). The variations of lattice parameters and Mn–F distances induced by applied pressure are displayed in Table 3. Both codes lead to very similar results.

**Table 3:** Evolution of Lattice Parameters and Mn–F Distances for CsMnF_4_ with Pressure Calculated with VASP (First Row) and CRYSTAL (Second Row) Codes^a^

*P* (GPa)	Symmetry	*a* (Å)	*c* (Å)	*R*_Z_ (Å)	*R*_X_ (Å)	*R*_Y_ (Å)
0	*P*4/*n*	8.029	6.401	1.829	1.871	2.189
		7.961	6.347	1.818	1.877	2.161
10	*P*4/*n*	7.649	5.970	1.802	1.858	2.058
		7.632	5.949	1.794	1.868	2.042
20	*P*4/*n*	7.442	5.763	1.784	1.839	1.997
		7.437	5.759	1.779	1.852	1.985
30	*P*4/*n*	7.282	5.632	1.771	1.821	1.957
		7.285	5.637	1.766	1.835	1.947
40	*P*4/*n*	7.146	5.545	1.760	1.806	1.925
		7.154	5.557	1.757	1.819	1.918
40	*P*4	7.172	5.430	1.755	1.833	1.939
		7.181	5.444	1.751	1.844	1.924
50	*P*4	6.988	5.532	1.745	1.853	1.851
		7.106	5.283	1.733	1.869	1.885

a Below *P* = 40 GPa the equilibrium structure is that observed at ambient pressure (space group *P*4/*n*) with MnF_6_^3–^ units in the high-spin configuration (*S* = 2). At *P* = 40 GPa the *P*4/*n* structure becomes unstable giving rise to a new equilibrium structure described by the *P*4 space group.

**Figure 6:**
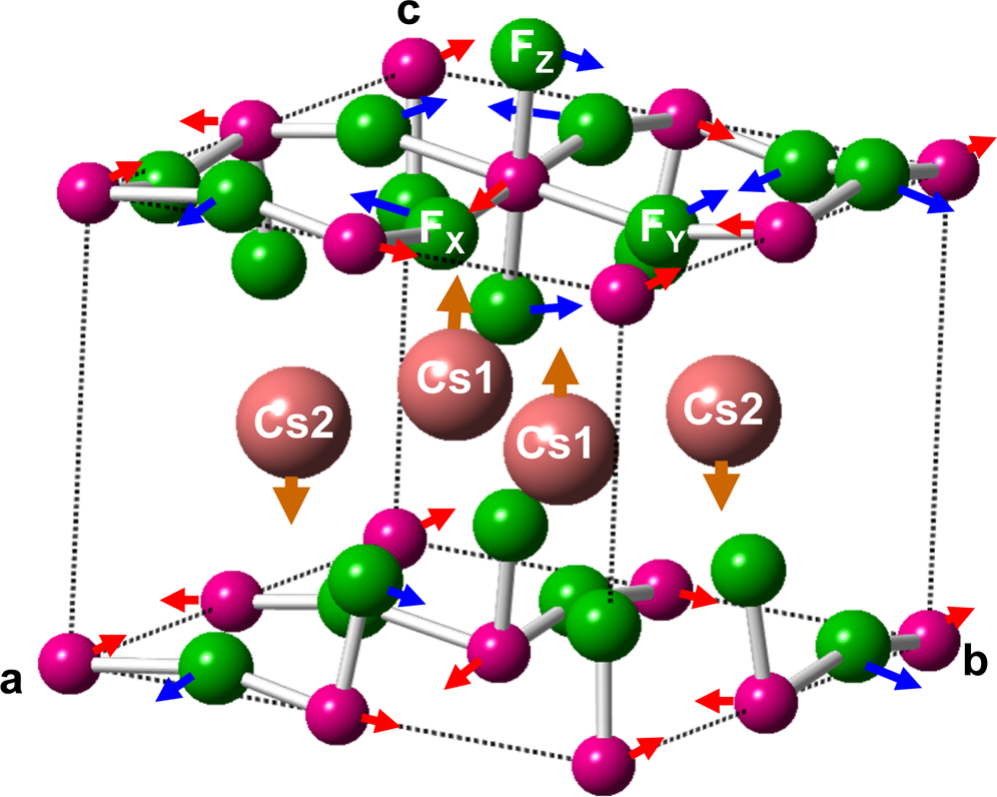
Qualitative picture of the small distortions produced by the unstable a_2g_ modes on the *P*4/*n* structure of CsMnF_4_ at *P* = 37.5 GPa producing the *P*4 phase, where the MnF_6_^3–^ units have triclinic *C*_1_ symmetry.

Nevertheless, at *P* = 40 GPa the present calculations indicate that the *P*4/*n* structure becomes unstable, being slightly distorted to another one with a *P*4 space group (Figure 6). In both phases, the ground state of MnF_6_^3–^ units is found to correspond to the high-spin configuration involving *S* = 2.

It should be noted that in the *P*4 phase at *P* = 40 GPa the MnF_6_^3–^ complexes have local triclinic symmetry (point group *C*_1_), with small distortions in the Mn–F distances and in the F–Mn–F angles with respect to the *P*4/*n* structure at the same pressure (see Figure S1 of the Supporting Information). For simplicity, Tables 3 and 5 show the average values of the Mn–F distances in the 3 local directions X, Y, and Z of the complexes, although the calculations of the d–d transitions have been carried out with the optimized geometries.

It should be also noted that within the *P*4/*n* phase, the length reduction due to pressure is much bigger for the long bond of MnF_6_^3–^ than for the two others. For instance, in the range 0–40 GPa, *R*_Y_ decreases by 0.25 Å while *R*_X_ and *R*_Z_ are reduced only by 0.03 and 0.07 Å, respectively (Table 3). In other words, pressure helps the geometry of the MnF_6_^3–^ complex to become closer to the octahedral one as *R*_Y_ – *R*_Z_ goes from 0.36 Å at ambient pressure to only 0.16 Å when *P* = 40 GPa. This behavior is very similar to that found for the hybrid layered perovskite (C_2_H_5_NH_3_)_2_CdCl_4_ doped with Cu^2+^ and plays a key role for explaining the shifts undergone by d–d transitions under pressure,^71^ a question analyzed in the next subsection.

Concerning the *P*4/*n* → *P*4 phase transition at *P* = 40 GPa, calculations lead to an enthalpy difference Δ*H* = H(*P*4) – H(*P*4/*n*) equal only to −0.023 eV and −0.030 eV from VASP and CRYSTAL codes, respectively. It is worth noting that according to calculations, the three Mn–F distances of MnF_6_^3–^ are only slightly influenced by the phase transition. Indeed, the variations undergone by *R*_X_, *R*_Y_, and *R*_Z_ at *P* = 40 GPa on changing from *P*4/*n* to *P*4 are smaller than 1.4% (Table 3). This fact thus suggests that the *P*4/*n* → *P*4 phase transition does not produce significant jumps in optical transitions, a matter treated in the next subsection.

Although the present calculations lead to an electronic ground state of MnF_6_^3–^ units coming from the ^5^E_g_(t^3^e^1^) high-spin configuration in *O*_*h*_ symmetry, we have also paid attention to determine the enthalpy of the lowest state emerging from the ^3^T_1g_(t^4^e^0^) low-spin configuration in *O*_*h*_ symmetry. The difference of the enthalpy per molecule, Δ*H*, between low-sping (*S* = 1) and high-spin (*S* = 2) configurations derived for both the *P*4/*n* and *P*4 structures at 40 and 50 GPa is displayed in Table 4. The calculated Δ*H* values by means of VASP and CRYSTAL codes are all in the range 0.45–0.68 eV. Therefore, the assumption of a transition from *S* = 2 to *S* = 1 induced by pressure at about 40 GPa^17^ is highly unlikely.

**Table 4:** Difference of the Enthalpy Per Molecule, Δ*H* (in eV), between Low-Spin (*S* = 1) and High-Spin (*S* = 2) Configurations Calculated for Both *P*4/*n* and *P*4 Phases at Pressures of *P* = 40 and 50 GPa^a^

*P*	Phase	Δ*H*
40	*P*4/*n*	0.63
		0.53
40	*P*4	0.65
		0.56
50	*P*4/*n*	0.48
		0.39
50	*P*4	0.45
		0.50

a Results obtained using VASP (first line) and CRYSTAL (second line) codes are both displayed.

### Spin-Allowed d–d Transitions in CsMnF_4_ under Pressure

Considering the equilibrium geometries derived for CsMnF_4_ at different pressures in Table 3, we have calculated in a further step the evolution of spin-allowed d–d transitions for pressures up to 40 GPa using both VASP and CRYSTAL codes. Results displayed in Table 5 correspond to the *P*4 phase for *P* = 40 GPa and to the *P*4/*n* phase for the rest of the pressures. Both codes lead to similar results.

**Table 5:** Calculated Energies (in eV) of Four Spin-Allowed d–d Transitions of MnF_6_^3–^ Units in CsMnF_4_ for Different Pressures, *P* (in GPa)^a^

*P*	Symmetry	*xy* → *x*^2^–*z*^2^	*yz* → *x*^2^–*z*^2^	*xz* → *x*^2^–*z*^2^	3*y*^2^–*r*^2^ → *x*^2^–*z*^2^
0	*P*4/*n*	2.83	2.48	2.18	1.84
		2.81	2.60	2.26	1.92
10	*P*4/*n*	2.94	2.53	2.33	1.55
		2.84	2.48	2.28	1.54
20	*P*4/*n*	3.02	2.63	2.45	1.44
		2.93	2.54	2.38	1.48
30	*P*4/*n*	3.10	2.73	2.57	1.38
			2.61	2.47	1.43
40	*P*4	2.99	2.72	2.56	1.47
		3.02	2.56	2.47	1.43

a First and second lines show the results obtained through VASP and CRYSTAL + ADF codes, respectively. The energies of all transitions are in eV. Note that data for *P* = 40 GPa correspond to the stable *P*4 phase, while for lower pressures the equilibrium structure corresponds to *P*4/*n*. Transitions are described though the dominant character of the involved orbitals. The influence of the internal electric field, E_R_(**r**), on the calculated d–d transitions is systematically taken into account.

The results presented in Table 5 show the existence of four allowed d–d transitions, in accord with the orthorhombic symmetry of MnF_6_^3–^ units. As expected from Figure 3 the lowest d–d excitation actually corresponds to |3y^2^–*r*^2^⟩ → |*x*^2^–*z*^2^⟩ for all pressures.

The experimental spectrum of CsMnF_4_ obtained at room temperature and ambient pressure (Figure 2) was assumed to involve only three spin-allowed d–d transition with energies^17^*E*_0_ = 1.80 eV, *E*_1_ = 2.26 eV, and *E*_2_ = *E*_3_ = 2.80 eV. According to Table 5, such transitions can now reasonably be assigned as |3y^2^–*r*^2^⟩ → |*x*^2^–*z*^2^⟩, |*xz*⟩ → |*x*^2^–*z*^2^⟩, and |*xy*⟩ → |*x*^2^–*z*^2^⟩, respectively. In the poorly resolved experimental spectrum (Figure 2), the |*yz*⟩ → |*x*^2^–*z*^2^⟩ transition, calculated at about 2.55 eV, is not well seen likely due to the width of the |*xz*⟩ → |*x*^2^–*z*^2^⟩ and |*xy*⟩ → |*x*^2^–*z*^2^⟩ transitions as well as to the presence of spin-forbidden transitions in the spectrum.

**Figure 7:**
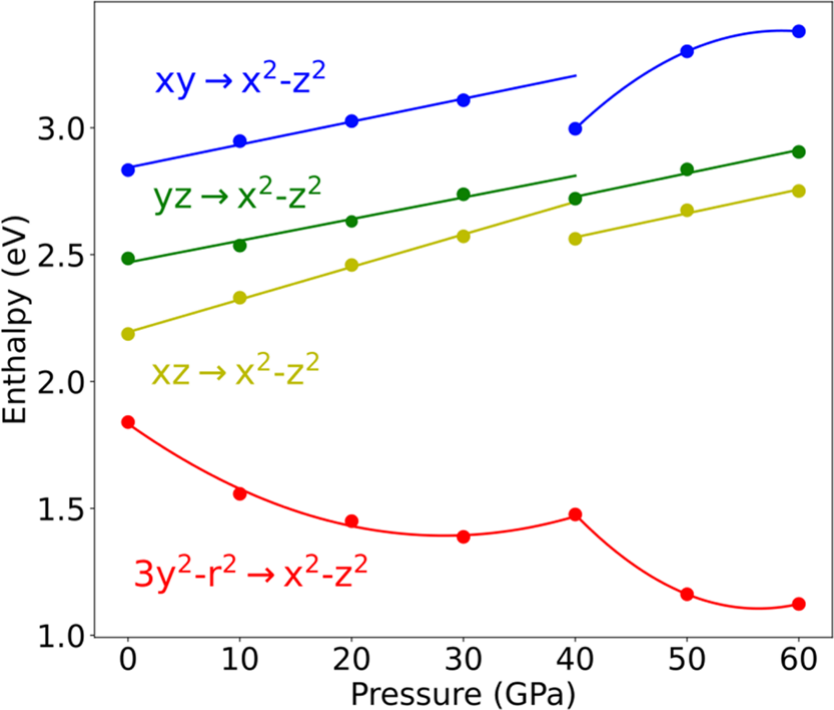
Variation of the four d–d transition energies of CsMnF_4_ in the 0–60 GPa pressure range. Values were calculated with the VASP code, but similar variations were obtained with CRYSTAL + ADF codes.

The calculated evolution of four spin-allowed d–d transitions with pressure (Table 5) is depicted in Figure 7. It should be noted that the first |3y^2^–*r*^2^⟩ → |*x*^2^–*z*^2^⟩ transition experiences a drastic red shift with pressure as it moves from 1.84 eV at zero pressure to 1.47 eV at *P* = 40 GPa. By contrast, the three transitions associated with t_2g_ orbitals in *O*_*h*_ symmetry undergo a moderate blue-shift under pressure. Interestingly, the energy difference, Δ(*xy*,*xz*), between |*xy*⟩ → |*x*^2^–*z*^2^⟩ and |*xz*⟩ → |*x*^2^–*z*^2^⟩ transitions is reduced significantly on passing from zero (Δ(*xy*,*xz*) = 0.65 eV) to 40 GPa (Δ(*xy*,*xz*) = 0.43 eV). At the same time, the energies of the two transitions associated with |*xz*⟩ and |*yz*⟩ orbitals become equal within ∼0.1 eV at *P* = 40 GPa such as it is shown in Table 5. Therefore, the very broad band observed at around 40 GPa that peaked at 2.5 eV likely involves the unresolved contributions of three transitions associated with the three t_2g_ orbitals in *O*_*h*_ symmetry.

The evolution of four spin-allowed d–d transitions of CsMnF_4_ under pressure (Table 5 and Figure 7) is consistent with the calculated variations undergone by the Mn–F distances (Table 3). Indeed, we have seen that pressure favors a geometry of the MnF_6_^3–^ complex progressively closer to the octahedral one thus reducing Δ(*xy*,*xz*) as well as the energy of the |3y^2^–*r*^2^⟩ → |*x*^2^–*z*^2^⟩ transition. Along this line, we have found that *R*_Y_ related to the softest Mn–F bond is much more reduced by pressure than *R*_X_ or *R*_Z_ (Table 3), and thus, we can reasonably expect a red-shift for that transition under pressure. This behavior is fully similar to that found in hybrid layered perovskites^69^ like (CH_3_NH_3_)_2_CuCl_4_, (C_2_H_5_NH_3_)_2_CuCl_4_, or (C_2_H_5_NH_3_)_2_CdCl_4_:Cu^2+^.

In a further step, it is necessary to disclose the influence of the internal field  on the optical transitions of CsMnF_4_ as it plays an important role in the case of inorganic layered perovskites like K_2_CuF_4_ or Cs_2_AgF_4_.^6,8,23^ For clarifying this issue, we have calculated in a first step the energies of four d–d transitions considering only the *isolated* MnF_6_^3–^ unit at the equilibrium geometry while in a second step we have added the influence of the electrostatic potential, V_R_(**r**). Results have been derived for *P* = 0 and 20 GPa and are shown in Table 6. It can be noticed that the addition of **E**_R_(**r**) produces an important shift of about 0.5 eV on the energy of the lowest d–d transition, a result qualitatively consistent with the shape of V_R_(**r**) (Figure 4). Indeed (−e)V_R_(**r**) tends to decrease the energy of orbitals, such as |3y^2^–*r*^2^⟩, lying in the layer plane thus enhancing the |3y^2^–*r*^2^⟩ → |*x*^2^–*z*^2^⟩ transition energy. A similar situation is encountered in K_2_CuF_4_ or Cs_2_AgF_4_.^23^

**Table 6:** Influence of the Internal Electric Field, **E**_R_(**r**), on the Energy (in eV) of Four d–d Transitions Corresponding to MnF_6_^3–^ Units in CsMnF_4_ for Two Pressures, *P* = 0 and 20 GPa^a^

Pressure	*xy* → *x*^2^–*z*^2^	*yz* → *x*^2^–*z*^2^	*xz* → *x*^2^–*z*^2^	3*y*^2^–*r*^2^ → *x*^2^–*z*^2^
0	2.62	2.51	2.23	1.44
	2.81	2.60	2.26	1.92
20	2.69	2.54	2.38	0.95
	2.93	2.54	2.38	1.48

a The first row corresponds to calculated values with CRYSTAL + ADF codes on an isolated MnF_6_^3–^ unit at the equilibrium geometry corresponding to the applied pressure, while in the second row are shown the values derived including the electrostatic potential *V*_R_(**r**) in the calculation. Transitions are described though the dominant character of involved orbitals.

Finally, for the sake of clarity, we have performed an analysis of three contributions responsible for the energy *E*_0_ of the first spin-allowed transition |3y^2^–*r*^2^⟩ → |*x*^2^–*z*^2^⟩ of CsMnF_4_ at zero pressure. According to the present discussion, we divide the calculation in 3 steps: (1) We consider an isolated MnF_6_^3–^ unit in the tetragonal geometry of the parent phase, obtaining a value *E*_0_ = 0.89 eV. (2) In a second step, we still keep the isolated MnF_6_^3–^ unit but in the final *P*4/*n* geometry where MnF_6_^3–^ exhibits a practical orthorhombic symmetry with *R*_Y_ – *R*_X_ = 0.31 Å, obtaining *E*_0_ = 1.44 eV. (3) In the final step, we include the shift due to the internal V_R_(**r**) potential on the energy of the transition, obtaining a value *E*_0_ = 1.92 eV. Therefore, the contributions of both the orthorhombic distortion and V_R_(**r**) enhance the *E*_0_ value by ∼1 eV.

### Survey of Other Compounds Containing MnF_6_^3–^ Units

Thanks to the analysis carried out in preceding sections on CsMnF_4_ we can now gain better insight into the different optical properties at zero pressure displayed by other fluorides involving Mn^3+^. In a first step we pay attention to the Na_3_MnF_6_ compound^12,13^ where the metal–ligand distances are *R*_*z*_ = 2.018 Å, *R*_*x*_ = 1.862 Å, and *R*_*y*_ = 1.897 Å. Accordingly, in this case the longest metal–ligand distance is along the Z axis, the orthorhombicity is small (*R*_*y*_ – *R*_*x*_ = 0.035 Å), and the HOMO practically equal to |3*z*^2^–*r*^2^⟩. The energies of allowed d–d transitions measured experimentally^12^ and derived by means of first-principles calculations^13^ are reported in Table 7. It can be seen that the transitions |t_i_⟩ → |3*z*^2^–*r*^2^⟩ (t = *xy*, *xz*, *yz*) all are in the 2–3 eV range, as has been found for CsMnF_4_. However, the energy of the first |3*z*^2^–*r*^2^⟩ → |*x*^2^–*y*^2^⟩ transition is practically half the value *E*_0_ = 1.9 eV obtained for CsMnF_4_, thus involving a remarkable difference. A similar situation is encountered when looking at the first transition of K_3_MnF_6_ or Cs_3_MnF_6_ where *E*_0_ = 1.1 eV.^72,57^

**Table 7:** Energies of Spin-Allowed d–d Transitions for MnF_6_^3–^ Units in Na_3_MnF_6_ Derived at Ambient Pressure^a^

	3*z*^2^–*r*^2^ → *x*^2^–*y*^2^	*xy* → *x*^2^–*y*^2^	*xz* → *x*^2^–*y*^2^	*yz* → *x*^2^–*y*^2^	ref.
Experimental	1.04	2.18	2.38	2.38	(54)
		2.17	2.38	2.58	(12)
Calculated	0.71	2.11	2.27	2.32	(13)

a In this compound the metal–ligand distances^12^ are *R*_*z*_ = 2.018 Å, *R*_*x*_ = 1.862 Å, and *R*_*y*_ = 1.897 Å giving rise to a small orthorhombicity (*R*_*y*_ – *R*_*x*_ = 0.035 Å) and a HOMO practically equal to |3*z*^2^–*r*^2^⟩. Transition energies (in eV) have been obtained from both experiments and calculations.

There are two main reasons behind a *E*_0_ value around 1 eV for Na_3_MnF_6_. On one hand, the orthorhombicity of MnF_6_^3–^ units in this compound is 1 order of magnitude smaller than that found for CsMnF_4_. On the other hand, Na_3_MnF_6_ is not a typical layered compound like K_2_CuF_4_ or CsMnF_4_ and the internal electric field, **E**_R_(**r**), has proven to induce shifts on d–d transitions not higher than 0.1 eV.^13^

It is worth noting now that a similar situation has been found when comparing fluoride compounds containing Cu^2+^, such as KZnF_3_:Cu^2+^, K_2_ZnF_4_:Cu^2+^, and K_2_CuF_4_. As KZnF_3_ is cubic there is a static JT effect in KZnF_3_:Cu^2+^, with an unpaired electron in |*x*^2^–*y*^2^⟩.^73−75^ Due to the cubic symmetry of the host lattice, **E**_R_(**r**) has no effect on the first d–d transition, |3*z*^2^–*r*^2^⟩ → |*x*^2^–*y*^2^⟩, whose energy is *E*_0_ = 0.40 eV.^74,75^ As K_2_ZnF_4_ is a layered compound where the shape of V_R_(**r**) is similar to that of Figure 4, the unpaired electron is forced to be in a |3*z*^2^–*r*^2^⟩ orbital by the action of V_R_(**r**), and consequently the |*x*^2^–*y*^2^⟩ → |3*z*^2^–*r*^2^⟩ transition energy is enhanced having a value *E*_0_ = 0.70 eV.^76,61,74^ Finally, as in K_2_CuF_4_ two adjacent CuF_6_^4–^ units share a common ligand, this favors an orthorhombic instability which still increases the *E*_0_ value up to 1.03 eV.^77,78,74,9^

### Magnetic Structure of CsMnF_4_: Influence of Pressure

**Figure 8:**
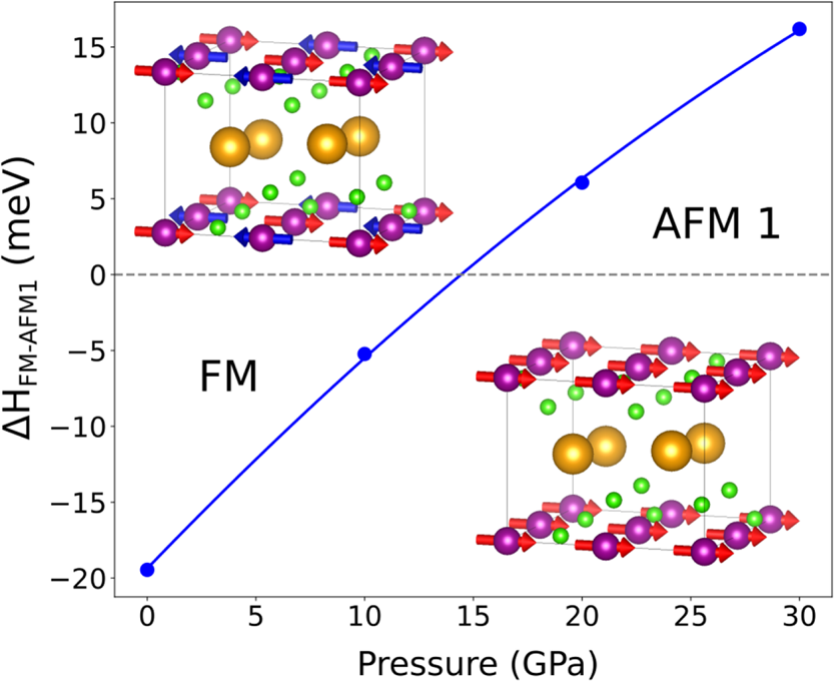
Evolution of magnetic ordering in CsMnF_4_ as a function of pressure. In the figure is depicted the enthalpy difference (given per formula unit, in meV) between ferromagnetic and antiferromagnetic ordering calculated for pressures up to 30 GPa where CsMnF_4_ is always in the *P*4/*n* structure.

As shown in Figure 8, the present calculations support that in CsMnF_4_ at ambient pressure layers are ferromagnetically ordered. This behavior, consistent with experimental data, is the same found for layered compounds like K_2_CuF_4_ or Cs_2_AgF_4_ where the M–F–M angle, θ, (M = Cu, Ag) is 180° due to the absence of buckling at the *Cmca* equilibrium structure.^9,22^ The ferromagnetism displayed by K_2_CuF_4_ or Cs_2_AgF_4_ is surprising as tetragonal K_2_MnF_4_ and K_2_NiF_4_ compounds, where the θ angle is also equal to 180°, exhibit an AFM ordering the same found for KMnF_3_ and KNiF_3_ perovskites.^79^ Very recently, the ferromagnetism in K_2_CuF_4_ and Cs_2_AgF_4_ at the *Cmca* equilibrium structure has proved to come from the orthorhombic distortion undergone by MF_6_^4–^ units (M = Cu, Ag), which in turn is actually responsible for the orbital ordering in these compounds.^22^ Indeed, in the *I*4/*mmm* parent phase of K_2_CuF_4_ and Cs_2_AgF_4_, involving tetragonal MF_6_^4–^ units (M = Cu, Ag), the calculations lead to an AFM ordering similar to that observed for K_2_MnF_4_ or K_2_NiF_4_ at ambient pressure.

**Figure 9:**
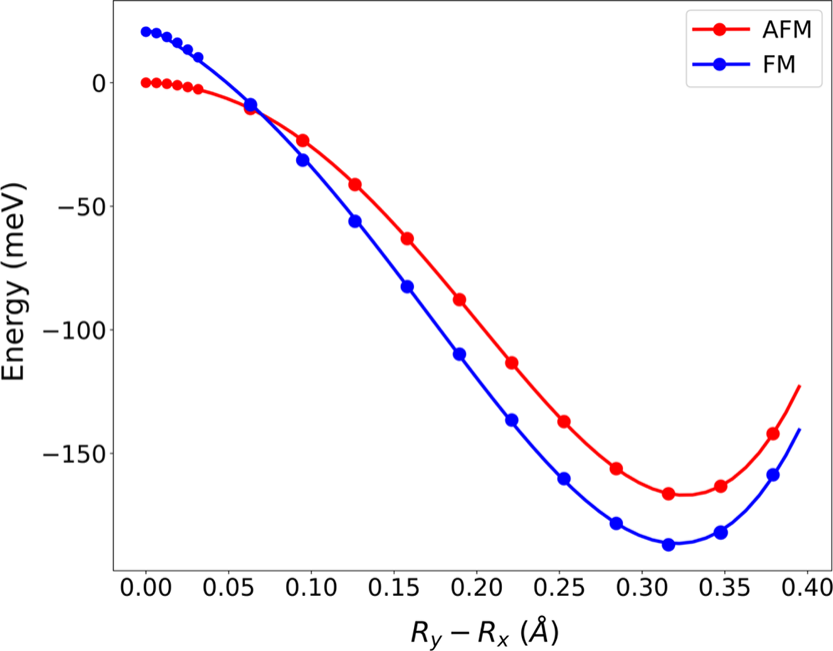
Energy (given per formula unit) of CsMnF_4_ obtained by single-point calculations throughout the antiferrodistortive distortion from the parent tetragonal *P*4/*nmm* phase (*R*_Y_ – *R*_X_ = 0) to the *P*4/*n* structure where *R*_Y_ – *R*_X_ = 0.32 Å at equilibrium. In this process, the AFM and FM ordering in the layers of CsMnF_4_ are both considered.

We verified that a similar situation holds for CsMnF_4_. Indeed, in the *P*4/*nmm* parent phase, where *R*_*y*_ = *R*_*x*_ we also find that the layers of CsMnF_4_ are AFM ordered although the FM ordering has an energy that is only 20.6 meV above (Figure 9). Moreover, on passing progressively at zero pressure from the *P*4/*nmm* parent phase to the equilibrium *P*4/*n* structure, CsMnF_4_ easily becomes ferromagnetic following the increase of the orthorhombic distortion on the MnF_6_^3–^ units, as shown in Figure 9.

It is worth noting that, on passing from *R*_Y_ – *R*_X_ = 0 to *R*_Y_ – *R*_X_ = 0.32 Å, the orthorhombic distortion implies an energy gain of 206.7 and 166.7 meV for the FM and AFM ordering, respectively (Figure 9). These values are thus 1 order of magnitude higher than the energy difference (20.6 meV) between both magnetic structures at the *P*4/*nmm* parent phase. This simple result just stresses that vibronic interactions, which are behind the orthorhombic MnF_6_^3–^ units at equilibrium, play an important role for explaining the magnetic structure in CsMnF_4_.

In K_2_CuF_4_ and Cs_2_AgF_4_, the shift from AFM to FM ordering when *R*_*y*_ – *R*_*x*_ increases has proved to arise from deep changes in chemical bonding in the MF_6_^4–^ units (M = Cu, Ag). Indeed, an increase of the orthorhombicity enhances the charge, *q*(*R*_X_), transferred to the closest ligands, placed at *R*_X_ from the cation, at the expense of two ligands at *R*_Y_ whose charge, *q*(*R*_Y_), is drastically reduced, being null at equilibrium.^22^ As the AFM contribution to the exchange constant depends on *q*(*R*_X_) × *q*(*R*_Y_), this fact favors the shift to a FM phase.^22^

When pressure increases, the results of the present calculations (Figure 8) reveal that above a pressure of 15 GPa the layers of CsMnF_4_ should be AFM ordered. Experimental data with pressures up to 4 GPa obtained by Ishizuka et al.^80^ show that the critical temperature, *T*_c_, decreases with pressure, a fact qualitatively consistent with results gathered in Figure 8. Along this line, recent GGA+U calculations by Behatha et al.^81^ also find a transition from the ferromagnetic to the AFM ordering although at a lower pressure of 2.4 GPa.

Two relevant facts are behind the pressure-induced shift from FM to AFM ordering in CsMnF_4_ shown in Figure 8. On one hand, there is a significant reduction of the orthorhombicity (Table 3). Indeed, while *R*_*y*_ – *R*_*x*_ = 0.32 Å at zero pressure, it becomes 44% smaller under a pressure of 15 GPa. On the other hand, the Mn–F–Mn angle in CsMnF_4_ changes from θ = 162.6° at zero pressure to θ = 153.4° at *P* = 15 GPa. Although in this process θ changes only by 5.7%, we have to recall that RbMnF_4_ at zero pressure (space group P2_1_/a) is AFM with a very low transition temperature (4 K) and an angle θ equal to 148°.^19^

## Conclusions

The existence of an orthorhombic instability plays a central role in understanding the optical and magnetic properties of layered compounds like CsMnF_4_ or K_2_CuF_4_. However, that instability is surprisingly not developed in CsFeF_4_ whose structure belongs to the *P*4/*nmm* space group and the FeF_6_^3–^ units are essentially tetragonal with *R*_*x*_ = *R*_*y*_.

According to eq 3, a negative force constant requires the admixture of the electronic ground state, Ψ_g_(r), with an excited state, Ψ_n_(r), via operator V(**r**) reflecting the electron–vibration coupling. As V(**r**) is a purely orbital operator, a necessary condition for having such an admixture and a force constant K < 0 is a matrix element ⟨Ψ_g_(**r**), *S*_g_| V(**r**) | Ψ_n_(**r**), *S*_*n*_⟩ different from zero, where *S*_g_ and *S*_*n*_ stand for the spin of ground and excited states, respectively. As FeF_6_^3–^ complexes in CsFeF_4_ are in a high-spin configuration this means a ground state with *S*_g_ = 5/2. If we now consider all excited states emerging from the d^5^ configuration of free Fe^3+^ ion, there are a total of 246 states.^43^ However, in such excited states the spin is at most *S*_*n*_ = 3/2 and thus none of them can be coupled to the ^6^A_1g_ state of the FeF_6_^3–^ unit. This spin barrier thus hampers the existence of orthorhombic instability in high-spin complexes of Fe^3+^ or Mn^2+^ ions.

That barrier does not exist for MnF_6_^3–^ complexes in CsMnF_4_ as the ^5^A_1g_ ground state can be mixed with excited ^5^B_1g_ through the orthorhombic b_1g_ mode. A similar situation holds for CuF_6_^4–^ units in layered lattices like K_2_CuF_4_, where the orthorhombic distortion has been shown to be directly responsible for its surprising FM behavior.^9,22^

By contrast, for tetragonal CrF_6_^3–^ complexes, there is also a hindrance against orthorhombic instability. Under *O*_*h*_ symmetry the ground state of CrF_6_^3–^ is ^4^A_2g_ which becomes ^4^B_1g_ in *D*_4*h*_. Among the 120 multiplets arising from the d^3^ configuration of free Cr^3+^ ion, only the states ^4^T_1g_ and ^4^T_2g_ have the same spin *S* = 3/2 as the ground state ^4^A_2g_ in *O*_*h*_ symmetry.^43^ Thus, a ^4^B_1g_ ground state in *D*_4*h*_ symmetry requires ^4^A_1g_ excited states for having a nonzero vibronic coupling associated with the b_1g_ mode. However, neither ^4^T_1g_ nor ^4^T_2g_ give rise to ^4^A_1g_ states under the *O*_*h*_ → *D*_4*h*_ symmetry reduction. No orthorhombic distortion is observed for the tetragonal K_2_MgF_4_ compound doped with Cr^3+^.^82,83^ A similar lack of excited states for tetragonal NiF_6_^4–^ units hampers the orthorhombic instability in K_2_NiF_4_.^22^

These reasons thus underline the origin of low-symmetry complexes widely observed for Cu^2+^ or Mn^3+^ systems and are not due to the Jahn–Teller effect. They are also behind the so-called plasticity property of compounds of Cu^2+^ and Mn^3+^ ions.^84^

We have seen that in CsMnF_4_ the internal electric field, **E**_R_(**r**), *increases* the value of the first d–d excitation by 0.5 eV, and a similar effect takes place in other layered compounds like K_2_CuF_4_.^23^ It is worth noting now that this behavior is not necessarily general, as in other systems **E**_R_(**r**) can give rise to an energy *reduction*. This is just what happens in the singular compound CaCuSi_4_O_10_, the basis for the historical Egyptian blue pigment,^85^ involving square-planar CuO_4_^6–^ complexes. In that compound the internal electric field produces a reduction of 0.9 eV in the highest d–d transition, which is thus directly responsible for its blue color.^86^

According to the present discussion, the behavior of d^4^ and d^9^ ions in tetragonal insulating lattices can hardly be ascribed to the Jahn–Teller effect. Along this line it is worth noting that even when such ions are initially located in a cubic symmetry there is not necessarily a static Jahn–Teller effect.^87^ For instance, in the Cu^2+^-doped cubic SrCl_2_ compound, the Cu^2+^ ion, initially replacing Sr^2+^, experiences a big off-center motion along ⟨001⟩ type directions driven by a force constant that becomes negative.^88,89^ A similar situation is found for Ag^2+^ and Ni^+^ in SrCl_2_ and also for SrF_2_:Cu^2+^ and CaF_2_:Ni^+^.^87^

Further work on the optical and magnetic properties of insulating transition metal compounds is now underway.

## Supporting Information Available

Additional computational details for both CRYSTAL17 (S1)25-27, and VASP codes (S2) and equilibrium geometries (S3); equilibrium geometries for *P*4/*n* phase at *P* = 0 GPa for *S* = 2, *P*4/*n* phase at *P* = 40 GPa for *S* = 2, and *P*4 phase at *P* = 40 GPa for *S* = 2 (PDF)
