# Supplementary material for: Understanding Pressure Effects on Structural, Optical, and Magnetic Properties of CsMnF4 and Other 3dn Compounds
Source: Inorg Chem. 2024 Jul 10;63(29):13231–43. doi: 10.1021/acs.inorgchem.4c00599 (PMC11271007; doi:10.1021/acs.inorgchem.4c00599)
Supplement: Supplementary file 1 — ic4c00599_si_001.pdf [file ic4c00599_si_001.pdf]

Supporting Information

Understanding Pressure Effects on Structural, Optical and Magnetic Properties of $CsMnF_4$ and Other $3d^n$ Compounds

Guillermo Santamaría †[a,b,c], Toraya Fernández-Ruiz†*[a], Juan María García-Lastra[d], Pablo García-Fernández[a], Inés Sánchez-Movellán[a], Miguel Moreno[a], José Antonio Aramburu[a]

[a] Departamento de Ciencias de la Tierra y Física de la Materia Condensada, Universidad de Cantabria, Avenida de los Castros s/n, 39005 Santander, Spain

[b] Donostia International Physics Center (DIPC), 20018 Donostia, Euskadi, Spain

[c] Laboratory of Chemistry of Novel Materials, University of Mons, Mons, Belgium

[d] Department of Energy Conversion and Storage, Technical University of Denmark. Anker Engelunds Vej. Building 301, 2800 Kgs. Lyngby, Denmark

* PhD Student Toraya Fernández-Ruiz ID https://orcid.org/0000-0001-8597-7133

E-mail: fernandezrt@unican.es

† Guillermo Santamaría and Toraya Fernández-Ruiz contributed equally to this work

S1. Computational details of Crystal17 calculations

We have used all electron triple-zeta polarized basis set of high quality developed by Peitinger et al.[1,2] for inorganic materials. For the exchange-correlation functional we used two one-parameter hybrid functionals, B1WC and PW1PW, which include 16% and 20% of Hartree-Fock exchange, respectively, and have provided accurate results for crystalline structures and properties of insulating systems containing transition metal ions.[3] The sampling of reciprocal space for the numerical integration within the Brillouin zone was 8x8x8 Monkhorst-Pack grid, which represents a distance between two consecutive k points of 0.144, 0.138 and 0.072 $Å^{-1}$ conforming to the three reciprocal space directions. For geometry optimizations the tolerance for energy change was 10^{-8} Hartree and for gradient and nuclei displacement were 0.0002 a.u.

Energies of the d-d optical transitions were calculated for each optimized geometry under pressure using a cluster approximation for a MnF_6^{3-} complex embedded in the electrostatic potential of the rest of lattice ions,[4] which was previously calculated through Ewald-Evjen summations.[5,6] calculations were performed by means of the Amsterdam density functional (ADF) code.[7] Hybrid B3LYP functional (25% of exact exchange)[8] combined with triple zeta polarized basis set have been employed in the simulations. The core electrons (1s-3p for Mn^{3+}, 1s for F^-) were kept frozen since they do not play a relevant role in the effects we focus on.

S2. Computational details of VASP calculations

The valence electrons were described by a plane-wave basis set with a 520 eV energy cutoff. The core electrons were described using the Projector Augmented Wave method (PAW)[9], in conjunction with pseudopotentials[10]. The valence configuration underlying the PAW potentials are thirteen, nine, and seven, respectively for Mn, F, and Cs. Geometry optimizations of the lattice parameters and atomic positions were carried out with the HSE06 functional[11], which incorporates 25% of Hartree-Fock (HF) exchange over a standard Perdew-Burke-Ernzerhof (PBE) Generalized Gradient Approximation (GGA). The convergence criterion for the electronic self-consistent loop was set to 1×10^{-6} eV, while atomic positions were relaxed until the forces per atom converged to less than 0.03 eV Å using the conjugate gradient algorithm[12]. The Brillouin zone was sampled using a 2×2×2 k-point mesh centered at the Γ point. The use of a relatively sparse k-point mesh is justified as the calculations carried out in VASP, that combine hybrid functionals and plane waves, are extremely computationally demanding. However, the results obtained are in an excellent agreement with those obtained with CRYSTAL17 and a much denser k-point mesh of 8 x 8 x 8.

Geometry optimizations under pressure from 0 to 60 GPa of pressure in 10 GPa increments were also performed with the VASP code using as initial geometries that provided by the CRYSTAL17 optimizations, significantly expediting the VASP convergence process. The initial occupancies of the orbitals were determined with a Gaussian

smearing of σ = 0.05. Calculations with high spin (S = 2) were carried out for each pressure, while for 40 GPa, an optimization with low spin (S = 1) was also included to study the potential spin crossover during the P4/n to P4 phase transition.

For the study of d-d optical transitions with VASP, we have used a different embedding by substituting three of the four Mn^{3+} atoms in the unit cell with Ga^{3+}, atoms with a d^{10} configuration and thus a symmetric electron density. The geometries used are the ones obtained from the optimization of the periodic $CsMnF_4$ system. The PAW method for the Ga atom encompassed thirteen valence electrons. In this case, the reciprocal space sampling was reduced to just the Γ point. The energies of the optical transitions were determined for each pressure ranging from 0 to 60 GPa, by calculating the difference between the Self-Consistent Field (SCF) enthalpy of the excited state and that of the ground state. The excited states were obtained by manually adjusting the occupations of the d-like Kohn-Sham orbitals using the ISMEAR keyword that determines how the partial occupancies are set for each orbital. Using ISMEAR = -2 the occupancies are read from the INCAR file (specify from FERWE and FERDO keywords for up and down spin channels respectively). These occupations remained fixed during the SCF minimization procedure.

S3. Equilibrium geometries of $CsMnF_4$

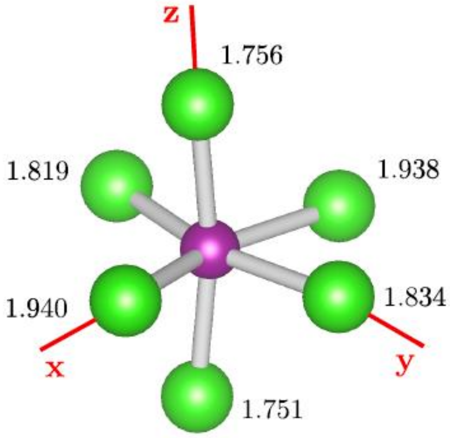

Figure S1. Triclinic geometry of the MnF_6^{3-} units obtained in the optimization with VASP of $CsMnF_4$ in P4 phase under a pressure of 40 GPa. Numeric values represent Mn-F distances in Å. All F-Mn-F differs from 90º.

The following tables show lattice parameters and atomic positions of CsMnF4 in the high-spin configuration S = 2. The geometries correspond to P4/n space group at P = 0 (Tables S1 and S2) and P = 40 GPa (Tables S3 and S4) as well as to P4 space group at P = 40 GPa (Tables S5 and S6). The results calculated with VASP are collected in Tables S1, S3 and S5 whereas the ones coming from CRYSTAL are gathered in Tables S2, S4 and S6.

Lattice vector	x (Å)	y (Å)	z (Å)
**a**	8.029	0.0	0.0
**b**	0.0	8.029	0.0
**c**	0.0	0.0	6.401
**Element**	**x**	**y**	**z**
**Mn**	0.0	0.0	0.0
**Mn**	0.5	0.5	0.0
**Mn**	0.5	0.0	0.0
**Mn**	0.0	0.5	0.0
**Cs**	0.75	0.25	0.5
**Cs**	0.25	0.75	0.5
**Cs**	0.25	0.25	0.448
**Cs**	0.75	0.75	0.552
**F**	0.230	0.997	0.048
**F**	0.770	0.003	0.952
**F**	0.270	0.503	0.048
**F**	0.730	0.497	0.952
**F**	0.503	0.230	0.048
**F**	0.497	0.770	0.952
**F**	0.997	0.270	0.048
**F**	0.003	0.730	0.952
**F**	0.968	0.975	0.281
**F**	0.032	0.025	0.719
**F**	0.532	0.525	0.281
**F**	0.468	0.475	0.719
**F**	0.525	0.968	0.281
**F**	0.475	0.032	0.719
**F**	0.975	0.532	0.281
**F**	0.025	0.468	0.719

Table S1. Lattice vectors a, b, c (in Å) and atomic positions (in fractional coordinates) derived from VASP calculations for the P4/n phase with S = 2 of $CsMnF_4$ at P = 0.

Lattice vector	x (Å)	y (Å)	z (Å)
a	7.961	0.0	0.0
b	0.0	7.961	0.0
c	0.0	0.0	6.347
Element	x	y	z
Mn	0.0	0.0	0.0
Mn	-0.5	-0.5	0.0
Mn	-0.5	0.0	0.0
Mn	0.0	-0.5	0.0
Cs	-0.25	0.25	-0.5
Cs	0.25	-0.25	-0.5
Cs	0.25	0.25	0.443
Cs	-0.25	-0.25	-0.443
F	0.232	-0.003	0.053
F	0.268	-0.497	0.053
F	-0.497	0.232	0.053
F	-0.003	0.268	0.053
F	-0.232	0.003	-0.053
F	-0.268	0.497	-0.053
F	0.497	-0.232	-0.053
F	0.003	-0.268	-0.053
F	-0.035	-0.024	0.282
F	-0.465	-0.476	0.282
F	-0.476	-0.035	0.282
F	-0.024	-0.465	0.282
F	0.035	0.024	-0.282
F	0.465	0.476	-0.282
F	0.476	0.035	-0.282
F	0.024	0.465	-0.282

Table S2. Lattice vectors a, b, c (in Å) and atomic positions (in fractional coordinates) derived from CRYSTAL calculations for the P4/n phase of CsMnF4 at P = 0.

Lattice vector	x (Å)	y (Å)	z (Å)
a	7.146	0.0	0.0
b	0.0	7.146	0.0
c	0.0	0.0	5.545
**Element**	**x**	**y**	**z**
Mn	0.0	0.0	0.0
Mn	0.5	0.5	0.0
Mn	0.5	0.0	0.0
Mn	0.0	0.5	0.0
Cs	0.75	0.25	0.5
Cs	0.25	0.75	0.5
Cs	0.25	0.25	0.412
Cs	0.75	0.75	0.588
F	0.241	0.973	0.090
F	0.259	0.528	0.090
F	0.528	0.241	0.090
F	0.972	0.259	0.090
F	0.759	0.028	0.909
F	0.741	0.473	0.909
F	0.473	0.759	0.909
F	0.028	0.741	0.909
F	0.942	0.949	0.301
F	0.558	0.551	0.301
F	0.551	0.942	0.301
F	0.949	0.558	0.301
F	0.058	0.051	0.699
F	0.442	0.449	0.699
F	0.449	0.058	0.699
F	0.051	0.442	0.699

Table S3. Lattice vectors a, b, c (in Å) and atomic positions (in fractional coordinates) derived from VASP calculations for the P4/n phase with S = 2 of CsMnF4 at P = 40 GPa.

Lattice vector	x (Å)	y (Å)	z (Å)
**a**	7.154	0.0	0.0
**b**	0.0	7.154	0.0
**c**	0.0	0.0	5.557
**Element**	**x**	**y**	**z**
**Mn**	0.0	0.0	0.0
**Mn**	0.5	-0.5	0.0
**Mn**	0.5	0.0	0.0
**Mn**	0.0	-0.5	0.0
**Cs**	-0.25	0.25	-0.5
**Cs**	0.25	-0.25	-0.5
**Cs**	0.25	0.25	0.412
**Cs**	-0.25	-0.25	-0.412
**F**	0.243	-0.028	0.091
**F**	0.257	-0.472	0.091
**F**	-0.472	0.243	0.091
**F**	-0.028	0.257	0.091
**F**	-0.243	0.028	-0.091
**F**	-0.257	0.472	-0.091
**F**	0.472	-0.243	-0.091
**F**	0.028	-0.257	-0.091
**F**	-0.057	-0.051	0.3
**F**	-0.443	-0.449	0.3
**F**	-0.449	-0.057	0.3
**F**	-0.051	-0.443	0.3
**F**	0.057	0.051	-0.3
**F**	0.443	0.449	-0.3
**F**	0.449	0.057	-0.3
**F**	0.051	0.443	-0.3

Table S4. Lattice vectors a, b, c (in Å) and atomic positions (in fractional coordinates) derived from CRYSTAL calculations for the P4/n phase with S = 2 of CsMnF4 at P = 40 GPa.

Lattice vector	x (Å)	y (Å)	z (Å)
**a**	7.172	0.0	0.0
**b**	0.0	7.172	0.0
**c**	0.0	0.0	5.430
**Element**	**x**	**y**	**z**
**Mn**	0.758	0.750	0.003
**Mn**	0.242	0.250	0.003
**Mn**	0.250	0.758	0.003
**Mn**	0.750	0.242	0.003
**Cs**	0.0	0.5	0.529
**Cs**	0.5	0.0	0.529
**Cs**	0.0	0.0	0.373
**Cs**	0.5	0.5	0.548
**F**	0.996	0.684	0.078
**F**	0.004	0.316	0.078
**F**	0.316	0.996	0.078
**F**	0.684	0.004	0.078
**F**	0.512	0.744	0.913
**F**	0.489	0.256	0.913
**F**	0.256	0.511	0.913
**F**	0.744	0.489	0.913
**F**	0.712	0.715	0.317
**F**	0.288	0.285	0.317
**F**	0.285	0.7120	0.317
**F**	0.715	0.288	0.317
**F**	0.822	0.817	0.705
**F**	0.178	0.183	0.705
**F**	0.183	0.822	0.705
**F**	0.817	0.178	0.705

Table S5. Lattice vectors a, b, c (in Å) and atomic positions (in fractional coordinates) derived from VASP calculations for the P4 phase with S = 2 of CsMnF4 at P = 40 GPa.

Lattice vector	x (Å)	y (Å)	z (Å)
**a**	7.181	0.0	0.0
**b**	0.0	7.181	0.0
**c**	0.0	0.0	5.444
**Element**	**x**	**y**	**z**
**Mn**	-0.244	-0.25	0.003
**Mn**	0.244	0.25	0.003
**Mn**	0.25	-0.244	0.003
**Mn**	-0.25	0.244	0.003
**Cs**	0.0	-0.5	-0.471
**Cs**	-0.5	0.0	-0.471
**Cs**	0.0	0.0	0.373
**Cs**	-0.5	-0.5	-0.455
**F**	-0.003	-0.314	0.077
**F**	0.003	0.314	0.077
**F**	0.314	-0.003	0.077
**F**	-0.314	0.003	0.077
**F**	-0.492	-0.256	-0.088
**F**	0.492	0.256	-0.088
**F**	0.256	-0.492	-0.088
**F**	-0.256	0.492	-0.088
**F**	-0.285	-0.283	0.317
**F**	0.285	0.283	0.317
**F**	0.283	-0.285	0.317
**F**	-0.283	0.285	0.317
**F**	-0.18	-0.184	-0.294
**F**	0.18	0.184	-0.294
**F**	0.184	-0.18	-0.294
**F**	-0.184	0.18	-0.294

Table S6. Lattice vectors a, b, c (in Å) and atomic positions (in fractional coordinates) derived from CRYSTAL calculations for the P4 phase with S = 2 of CsMnF4 at P = 40 GPa.

References

1. Peintinger, M. F.; Oliveira, D. V.; Bredow, T. J. Consistent Gaussian basis sets of triple-zeta valence with polarization quality for solid-state calculations. *J. Comput. Chem*. **2013**, 34, 451-459. DOI: 10.1002/jcc.23153
2. CRYSTAL basis sets. https://www.crystal.unito.it/basis-sets.php (accessed on May 29, 2022).
3. Bredow, T.; Gerson, A. Effect of exchange and correlation on bulk properties of MgO, NiO, and CoO. *Phys. Rev. B* **2000**, 61, 5194-5201. DOI: 10.1103/PhysRevB.61.5194
4. Moreno, M.; Aramburu, J. A.; Barriuso, M. T. Electronic Properties and Bonding in *Transition Metal Complexes: Influence of Pressure. In Optical Spectra and Chemical Bonding in Inorganic Compounds*; Mingos, D. M. P., Schönherr, T., Eds.; Structure

and Bonding, vol 106; Springer: Berlin, Heidelberg, **2004**; pp 127-152. DOI: 10.1007/b11309
5. Van Gool, W.; Piken, A. G. Lattice self-potentials and Madelung constants for some compounds. *J. Mater. Sci.* **1969**, 4, 95-104. DOI: 10.1007/BF00550650
6. Tosi, M. P. Cohesion of Ionic Solids in the Born Model. *Solid State Phys*. **1964**, 16, 1-120. DOI: 10.1016/S0081-1947(08)60515-9
7. te Velde, G.; Bickelhaupt, F. M.; Baerends, E. J.; Guerra, C. F.; van Gisbergen, S. J.; Snijders, J. D.; Ziegler, T. Chemistry with ADF. *J. Comput. Chem*. **2001**, 22, 931-967. DOI: 10.1002/jcc.1056
8. A. D. Becke, Density-functional thermochemistry. III. The role of exact exchange. *J. Chem. Phys.* **1993**, 98, 5648-5652. DOI: 10.1063/1.464913
9. Blöchl, P. E. Projector augmented-wave method. *Phys. Rev. B* **1994**, 50, 17953–17979. DOI: 10.1103/PhysRevB.50.17953
10. Kresse, G.; Joubert, D. From ultrasoft pseudopotentials to the projector augmented-wave method. *Phys. Rev. B* **1999**, 59, 1758–1775. DOI: 10.1103/PhysRevB.59.1758
11. Heyd, J.; Scuseria, G. E.; Ernzerhof, M. Hybrid functionals based on a screened Coulomb potential. *J. Chem. Phys*. **2003**, 118, 8207–8215. DOI: 10.1063/1.1564060
12. Press, W. H.; Teukolsky, S. A.; Vetterling, W. T.; Flannery, B. P. *Numerical Recipes: The Art of Scientific Computing*; Cambridge University Press: New York, **1986**.
